# Seeing the whole picture: integrated pre-surgery reports with PreOptique

**DOI:** 10.1186/s13326-019-0197-1

**Published:** 2019-03-04

**Authors:** Guillermo Vega-Gorgojo, Laura Slaughter, Martin Giese

**Affiliations:** 1 Department of Informatics, University of Oslo, Oslo, Norway; 2 Department of Signal Theory and Communications and Telematics, University of Valladolid, Valladolid, Spain

**Keywords:** Electronic health records, Systems integration, Biological ontologies, Ontology-based data access, Text analysis

## Abstract

**Background:**

Information technology has transformed the way healthcare is conducted. There is a deluge of patient data dispersed in different systems that are commonly not interoperable. As a result, access to patient data has become a major bottleneck for healthcare professionals that struggle to find the relevant information in a timely way and without missing critical clinical information.

**Results:**

We implemented PreOptique, a novel hybrid semantic and text-based system that was commissioned by a large hospital in Norway for providing integrated access to patient health records scattered over several databases and document repositories.

We use ontology-based data access (OBDA) for the seamless integration of the structured databases at the hospital through the Optique platform. We employ text analysis techniques to extract vital sign measures and clinical findings from patient documents.

PreOptique was developed and deployed at the hospital. This solution demonstrates how OBDA technology can provide integrated data access to disparate structured sources in healthcare, without requiring the replacement of existing databases. Unstructured clinical texts are also mined to extract patient findings, while the graphical user interface (GUI) provides a single access point that hides the underlying complexity of the system. We ran a usability study with 5 target users, obtaining a system usability score (SUS) of 86.0. Further, participants in the study stressed the simplicity of the GUI and the integration of data sources enabled by the system.

**Conclusions:**

This pilot study showcases the use of OBDA technology and text analysis to enable the integration of patient data for supporting clinical surgery operations. PreOptique is usable and can be easily employed by medical personnel to find patient data in a timely way**.**

## Background

Medical practice generates a deluge of patient data, including diagnostic codes, medication orders, laboratory test results, and medical imaging. Typically, several vendors supply systems to document and collect data related to patient care. Medical professionals use these systems for care planning and documentation purposes related to patient encounters with the healthcare system. When a patient is referred to a unit, the physician may order tests and imaging prior to seeing a patient. Healthcare professionals often use the system to access previously recorded clinical notes that can provide relevant background including diagnosis and even status regarding previous health-related assessments such as whether the patient has had a driver’s license revoked for medical reasons. Tracking all the data and relating it to the patient’s current status involves accessing the right system, evaluating the data that is there, checking date/time of documentation, assessing whether “this is the same or different incident” of a certain condition, and also contextual information like the type of medical encounter, e.g. a routine examination, or even the role of the reporter.

Despite the wealth of patient data and the omnipresence of healthcare systems, use of these systems does not necessarily equate to an increase of efficiency or improvements in quality of care [1]. One key issue is the lack of information exchange between systems which results in healthcare professionals using a great deal of time jumping between multiple different information technology (IT) systems in an attempt to tie together patient information. In this regard, [2] reports physicians’ frustration and professional dissatisfaction with electronic health record (EHR) systems due to “insufficient health information exchange”.

The interoperability of hospital IT systems is impacted by the many underlying policies, organizational constraints and culture, and understanding of hospital workflow reflected in the architecture and design of each system in use. To address this challenge, ontology-based data access (OBDA) technology can be used to seamlessly integrate structured data and information from these systems [3]. The role of an ontology is to define the principles ruling real-world entities and their interrelations, describing a domain’s inherent structure and behavior [4]. Semantic data integration involves the use of such a representation of entities and relationships to eliminate possible heterogeneities. The Optique project [5] has developed a number of tools and methods to support OBDA, including tools for enabling users to formulate queries using familiar vocabularies and conceptualizations, and integrating data spread across multiple distributed data sources.

### Ahus case study

Access to patient data is the chief complaint of the healthcare personnel at the day surgery unit in Akershus University Hospital (Ahus). Medical staff described an environment where they were allocated 20 min to check all patient information before surgery, but they were actually investing much longer times to prevent missing relevant information. We reproduce here some of their quotes:

*«We use too much time to search for information»*

*«We are not sure that we have checked all»*

*«We should rather spend the time on patients than on the computer systems»*

The underlying problem is that patient data is scattered across different hospital IT systems, i.e. the registry of medical encounters, the archive of clinical notes, the repository of patient measures, the registry of laboratory tests, and the pharmacy system. Furthermore, some of these systems lack the necessary functionalities to facilitate data access. As an example, Ahus staff can browse the clinical notes of a particular patient, but text search is not provided.

In this paper we present a novel hybrid semantic and text-based system that was commissioned by Ahus for providing integrated access to patient health records scattered in several databases and document repositories. The system makes use of the results from the Optique project and is based on reuse and extension of the OBDA tools available. The test case was the surgery planning process that involves surgeons, anesthesiologists, and nurses. The proposed system is named PreOptique (Pre-Op support with Optique). We showcase the benefits of this solution and present the results of a preliminary usability study.

## Implementation

### Ahus requirements

Ahus provides healthcare services to approximately 500,000 inhabitants in the county of Akershus, east of Oslo, Norway. Ahus is a mid-size/large hospital with 953 beds, 62,489 admitted patients, and 28,300 day patients registered in 2015. Patient data at Ahus is scattered across several systems – the main information systems employed by the day surgery unit are shown in Table 1. However, these systems are not integrated and access to patient data is not easy; for instance, there is no search facility for the DIPS document archive (DIPS stands for Distributed Information and Patient Data System in Hospital). As a result, medical professionals at Ahus complain about too much time spent searching patient information, and they fear missing critical information related to surgical preparation.

**Table 1:** Main information systems employed by the day surgery unit at Ahus

System	Provider	Type	Data description
DIPS	DIPS	Electronic health record system	SQL database with administrative data, medical encounters and patient measures
Metavision	Evry	Physician order entry system	SQL database with administrative data and patient measures Document archive of laboratory tests and medical images
DIPS archive	DIPS	Document repository	Document archive of unstructured clinical notes

We started a pilot project with the day surgery unit at Ahus aiming to improve the access to existing patient information. More specifically, Ahus requested support for the surgery planning process in which surgeons, anesthesiologists, and nurses collaboratively fill in a paper form with the operation plan. In a first stage, surgeon and patient agree on the surgery to be performed. Resources are then allocated and the preparations for the operation begin. There is a patient safety procedure before and after the operation to assess that the operation plan has been followed.

To complete the form, patient data has to be collected manually from the systems in Table 1, requiring a significant effort to find patient information and with concerns about missing critical pieces of information for the planned operation, e.g. an allergy. Therefore, the goal of the project was to provide an IT solution for supporting the surgery planning process with the following requirements:

- Integrate patient data coming from structured sources, i.e. the SQL databases of DIPS and Metavision.
- Perform text analysis of the DIPS document archive and provide access for the operation planning.
- Offer a single easy-to-use access point to patient data.
- Provide provenance information for every piece of patient data.
- Design a non-invasive solution, i.e. no replacement of the existing IT systems at Ahus.
- Provide adaptation to emerging user needs and new data sources such as the laboratory tests and medical images in Metavision that were not part of this pilot.

### The Optique platform

Optique is a solution for unlocking access to corporate data sources by enabling end users to formulate their information needs through an intuitive visual query interface [5]. The platform is based on OBDA technology [3] that provides an automated connection between complex information requirements and relational data stores. More specifically, an ontology is employed to describe the end users’ domain with familiar and comprehensible terms that are translated into queries over the data sources through a set of mappings.

Optique provides access to data in a non-invasive way, since the data sources do not need to be replaced or converted to another format. Instead, the platform manages the ontology and mappings, giving the illusion of a virtual integrated semantic store. In this way, a query formulation component such as OptiqueVQS [6] or PepeSearch [7] can directly plug in, enabling end users to pose ad hoc queries without requiring specialized IT skills.

Optique relies on open standards such as SPARQL [8] for querying the virtual triple store, OWL [9] for ontology specification, and R2RML [10] for the definition of mappings. Open standards avoid vendor lock-in situations and facilitate adaptation to diverse scenarios. For instance, Optique has been successfully deployed on Statoil’s corporate exploration and production data store [11], as well as on Siemens’ service centers for monitoring power plants [12, 13].

### Text analysis

While the Optique platform can be used to provide flexible access to structured data sources, it cannot be directly used with unstructured data. Instead, natural language processing (NLP) can be applied to analyze clinical text, the most common and abundant data type in the healthcare domain.

Text search engines [14] have become prevalent for dealing with unstructured data, e.g. Web search. Search engines maintain an index of the document corpus. Queries are evaluated against the index and results are then returned to the user. A query is typically composed of one or more keywords, although explicit phrases can also be supported. Potential answers are ranked using a similarity measure to estimate the relevance of a document for a query. The index is a data structure that maps terms to the documents that contain them, thus enabling fast query evaluation. Several parsing techniques are commonly applied in the construction of the index and in query evaluation, such as stemming (removal of variant endings from words), case folding (conversion to lowercase), or stopping (removal of common words such as *the*).

Besides regular text search, clinical documents can be mined to extract structured information about patients. This is typically done using NLP tools, which combine a range of linguistic, statistical and heuristic methods [15]. Deriving structured information from clinical text involves entity extraction algorithms that commonly employ medical vocabularies and ontologies such as SNOMED CT [16] to drive the entity extraction task. Difficulties in entity extraction include the presence of negating terms such as ‘no’ or ‘never’ [15]. cTAKES [17] is an example of an NLP tool for entity extraction from clinical text.

### PreOptique, integrated access to patient data

**Fig. 1:**
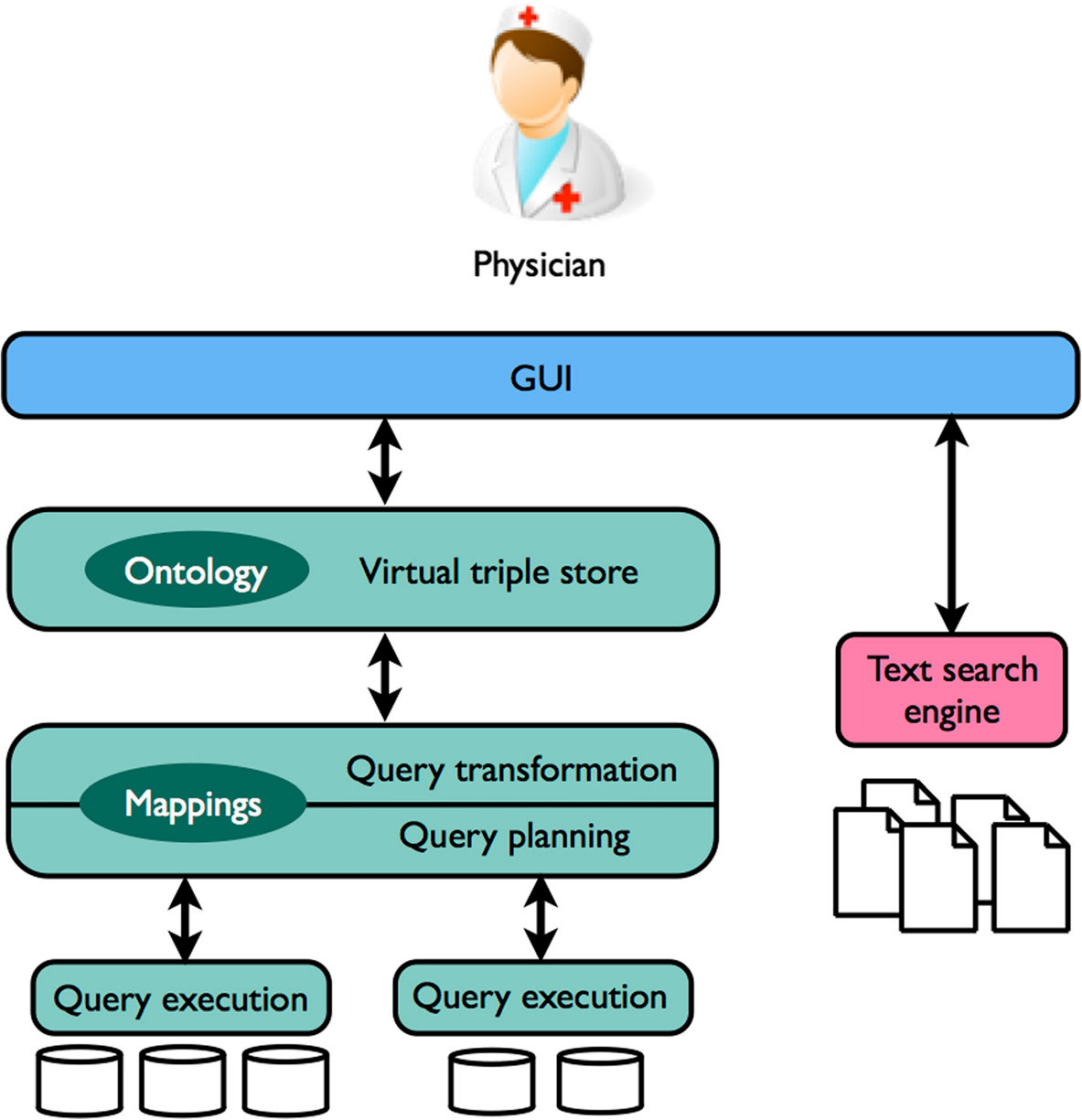
Logical architecture of PreOptique

We aimed to support the surgery planning process by offering a single easy-to-use access point to patient data without replacing the existing IT systems at Ahus. In order to comply with the requirements, we developed a hybrid semantic and text-based system named PreOptique. The logical architecture is sketched in Fig. 1 and has three main parts: the semantic backbone (teal color), the text search engine (pink), and the graphical user interface (GUI) that glues all the components and serves as entry point to the medical personnel (blue).

The semantic backbone is based on the Optique platform and deals with the integration of patient data coming from structured sources. The key artefact is the ontology that was developed to support this setting. The development of the ontology was thus driven by the database schemata of the structured sources employed at Ahus, i.e. DIPS and Metavision. We also used anonymized screenshots of the DIPS user interface to inform the design. In addition, we interviewed members of the medical staff at Ahus in order to gather the main limitations of the existing solution (see the reproduced quotes in Section 1.1). With these inputs, we identified that the main components the ontology should include: administrative patient data, medical diagnoses and diseases, healthcare encounters, data items such as documents, and measurements of vital signs.

For constructing the ontology we decided to reuse existing medical ontologies in the OBO Foundry suite [18]. OBO Foundry’s Basic Formal Ontology (BFO) [19] is an upper-level ontology that provides a common top-level structure to support the interoperability of the multiple domain ontologies. BFO forms the basis of numerous medical ontologies such as the NCBI organismal classification (NCBItaxon), Ontology of Medically Related Social Entities (OMRSE), Disease Ontology (DO), Ontology for General Medical Science (OGMS), Clinical Measurement Ontology (CMO), and the Information Artifact Ontology (IAO), or Relation Ontology (RO) – we reused classes from all these OFO Foundry ontologies.

The starting point was the definition of patient as a subclass of human being (NCBItaxon) that has a patient role (OMRSE). We borrowed properties from the FOAF vocabulary [20] to model basic patient data such as names, gender or image, while we defined local properties to represent the Norwegian social security number and the date of birth. We were therefore able to describe all the administrative patient data coming from DIPS and Metavision.

In Norway, medical diagnoses in the source DIPS EHR dataset are tagged using version 10 of the standard International Statistical Classification of Diseases and Related Health Problems diagnostic coding schema (ICD-10) [21]. DO is a domain ontology of human diseases based on BFO and organized from a clinical perspective of disease etiology and location [22]. DO is thus more closely aligned with how medical personnel work and think than ICD-10, so we decided to employ this ontology using disease from OGMS as the top concept. Since DO includes cross-references to ICD-10, we were able to map the medical diagnoses of patients in DIPS to DO terms. Alternatively, we could have employed the Monarchy Disease Ontology (MONDO) [23] that includes mappings to ICD-10 and DO.

We used health care encounter (OGMS) as the upper concept for modelling the various healthcare processes at Ahus. Specifically, we defined classes for every clinical operation specified in [24] since these codes are used in the source DIPS EHR dataset. We also prepared a taxonomy of data items (IAO) – reusing concepts from OGMS such as clinical finding and diagnosis – and arranging the different document types employed at Ahus, e.g. admission document. We extended RO to define object properties for connecting the different concepts, e.g. has disease as a subproperty of has disposition (RO), and datatype properties such as date of diagnosis. Overall, we had all the terms in the ontology for annotating the data items about medical encounters coming from DIPS.

For the measures of vital signs we selected scalar measurement datum (IAO) as the top concept and reused subtypes from CMO such as body temperature, pulse, or diastolic blood pressure. We also defined a local illness severity score concept (known as ASA in Norway), as requested by Ahus. Since patient measures can appear in DIPS, Metavision and even within unstructured clinical notes, it was important to track the source of data and we used the Provenance, Authoring, Versioning (PAV) ontology [25] for this purpose. This ontology was also used to trace the source of health care encounters and patient documents.

The ontology itself is formalized in OWL 2 QL, as required by the Optique platform. We give above a detailed description of the ontologies that were reused. Overall, we created 84 new classes, 36 new object properties, 12 new datatype properties, and 13 new annotation properties to accommodate patient data.

With the ontology in place, we created the mappings to the underlying databases, i.e. DIPS and Metavision, expressed in the W3C RDB2RDF Mapping Language (R2RML) [10]. This process was simplified by using the database schemata as the primary source for developing the ontology, so we already had in mind where the source relational data should be mapped in the ontology. A mapping assertion in R2RML consists of a SQL statement from the source database and a target definition of RDF triples constructed with the retrieved values from the source. The basic procedure comprised mapping every table in the databases to a class in the ontology, then mapping table columns to datatype properties, and finally mapping foreign keys to object properties. We defined the following scheme to mint the IRIs of the corresponding individuals: http://data.ahus.no/rd/{table_name}/{primary_key}.

Optique’s query transformation system, Ontop [26], was employed to rewrite user queries (expressed in terms of the ontology) into database queries using the ontology and the mappings. We used test instances of the databases of the pilot, i.e. DIPS and Metavision, in order to validate the mappings. More specifically, we assessed that the answers to user queries obtained with Ontop were consistent with the contents in the test database instances. In this fashion, Ontop exposes the underlying databases as a virtual RDF dataset that can then be queried using SPARQL. One obvious advantage of this approach is the integration of DIPS and Metavision without requiring the replacement or the modification of the relational data sources – this is illustrated in Section 3 with the measurements of vital signs coming from different sources.

Furthermore, the integration of additional domain knowledge into the system can better support the user needs at Ahus. To support our point, consider the following information need: *“find the names of the patients diagnosed with an intestinal disease”*. Posing a SQL query to satisfy this information need in DIPS is challenging because you need to know the list of all the ICD-10 codes corresponding to intestinal diseases. In contrast, this information need can be represented by the following SPARQL query:

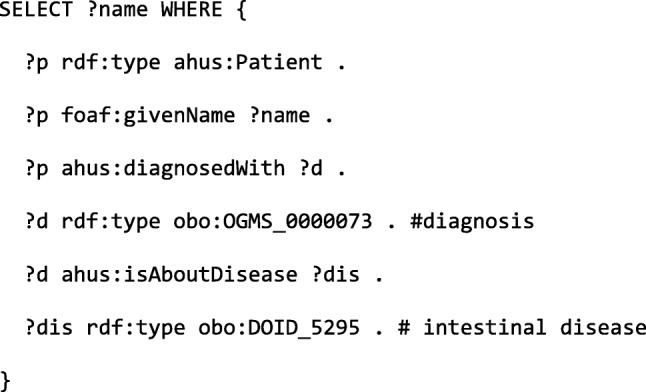



In this case, the use of DO allows the formulation of the query in a more abstract way and closer to the physicians’ needs. Note that a query builder like PepeSearch [7] can be plugged in to pose such SPARQL queries through a form-based web interface. We present in [27] a PepeSearch demo using a former version of the ontology developed at Ahus.

Regarding the collection of clinical notes, we used an open source search platform, Solr [28], that provides a scalable and flexible solution for querying unstructured text. We defined a document schema with metadata fields for document and patient identifiers, document types, and timestamps that was used to index the collection. Further configuration included stopping, case folding, and stemming for Norwegian text. With this set-up, Solr was ready to answer full-text searches over the collection of clinical notes, supporting queries with boolean expressions, phrases, fuzzy matching, and filters, e.g. to limit the results to a specific patient or to a document type. This alone was a significant improvement, since the DIPS archive at Ahus can only be browsed by document title.

Beyond supporting text search, we were required to automatically extract information about patient allergies, smoking habits, and measures of vital signs from the collection of clinical notes. This was a requirement due to the need of medical staff at Ahus to decrease time used in manual browsing of documents because the current archive is not searchable. We had access to a set of anonymized clinical notes in the Norwegian language for developing the information extraction module. The solution consisted on a set of Solr transformers that analyze every document in the collection and create new fields when an occurrence is found. As a first step, we defined fields in the document schema for accommodating the different types of information requested, e.g. blood_pulse_value, as well as additional fields for placing source text fragments, e.g. blood_pulse_info. This allowed the medical team to find a value extracted by a transformer as well as the original text fragment in the source document.

We used text tokenization, normalization, text expansion, and pattern matching of strings in the transformers. For every required information element we prepared a regular expression [29] for extracting the values. As an example, we defined the following regular expression for extracting a text snippet with blood pulse information (corresponding to the blood_pulse_info type that was defined as a first step):

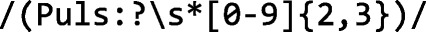



This regular expression is quite robust since it takes into account optional elements such as colon and whitespace. Further, pulse values require two or three digits. We constrained the range of possible values for every vital measure to limit errors. In other cases we invested a significant effort specifying alternative terms and abbreviations, e.g. the Norwegian word for temperature is *temperatur*, although physicians at Ahus also use the abbreviations *temp* and *t*. As a result, we defined regular expressions that capture alternative terms when necessary.

Extracting allergies and smoking habits from the text was more involved, requiring language analysis to deal with uncertainty and negation. We thus had to employ more varied and complex regular expressions to detect text snippets indicating positive, negative, or uncertain allergy status, as well as positive, negative, former, or uncertain smoker. We used the set of anonymized clinical notes to fine-tune the devised regular expressions. After importing a document to Solr, the set of transformers is run to extract the required information, creating appropriate fields with the values found. As an example, we provide below the set of regular expressions we crafted to detect a non-smoker and to extract the key text snippet (the part inside parenthesis):

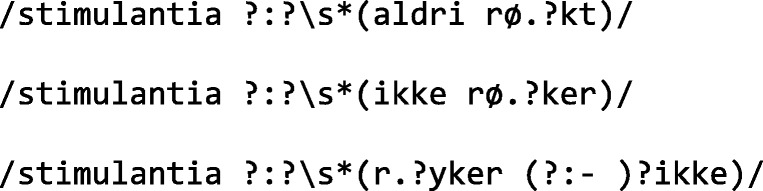



Finally, we aimed to design a GUI that would be easy to use and understandable to the medical personnel. We decided to employ a form-based GUI that is common in medical IT systems. The GUI includes a security module that grants access to authorized users. The medical personnel can then select a patient in the system and obtain all the information pulled from the different sources.

The integration of patient data is thus handled within the GUI (see Fig. 1). This component includes a semantic data access module for querying a triple store, as well as a text-based data access module for querying a Solr index. The former makes use of a bootstrapping SPARQL query for listing patients, while the rest of the queries are generic and can be applied to any individual (a patient, for example): one for obtaining the types; another for getting predicate-object lists; and another for getting subject-predicate lists referred to the target individual. Similarly, the text-based data access module is prepared to query the document index in order to find information contained in the clinical notes of a particular patient.

The GUI queries the data sources under demand and caches the responses obtained for performance and scalability reasons. The ontology drives the access to semantic data, depending on the type of information of interest. As an example, weight measurements of a patient can be obtained by looking for property ahus:personWeightMeasurement in the predicate-object list retrieved from the semantic data access module. Regarding clinical notes, the document schema plays a similar role to the ontology. This way, it is possible to query the Solr index to get patient documents containing weight measurements (as obtained in the text analysis stage).

**Fig. 2:**
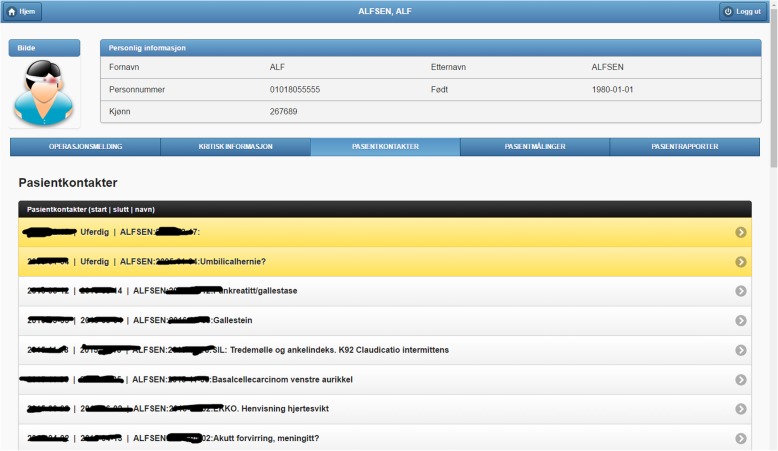
Excerpt of the medical encounters of a patient at Ahus. Each item includes a start date, a closing date and a title. Ongoing medical processes are shown in yellow

**Fig. 3:**
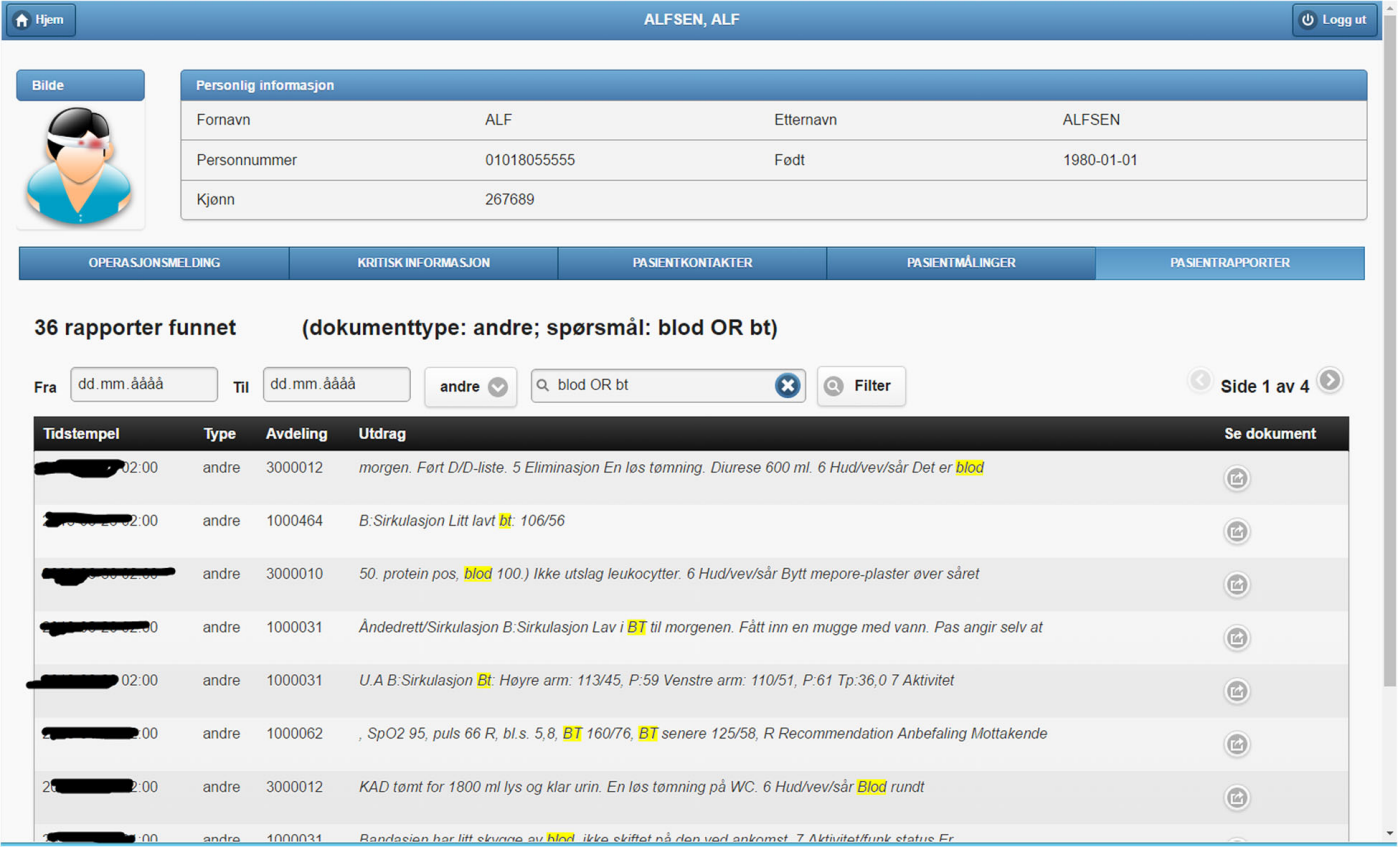
Example of a document search asking for documents of type “andre” (“other” in Norwegian) containing the keywords “blod” (“blood” in Norwegian) or “bt” (abbreviation of blood type). This screenshot shows that 36 documents have been found. Each item listed corresponds to a document found

**Fig. 4:**
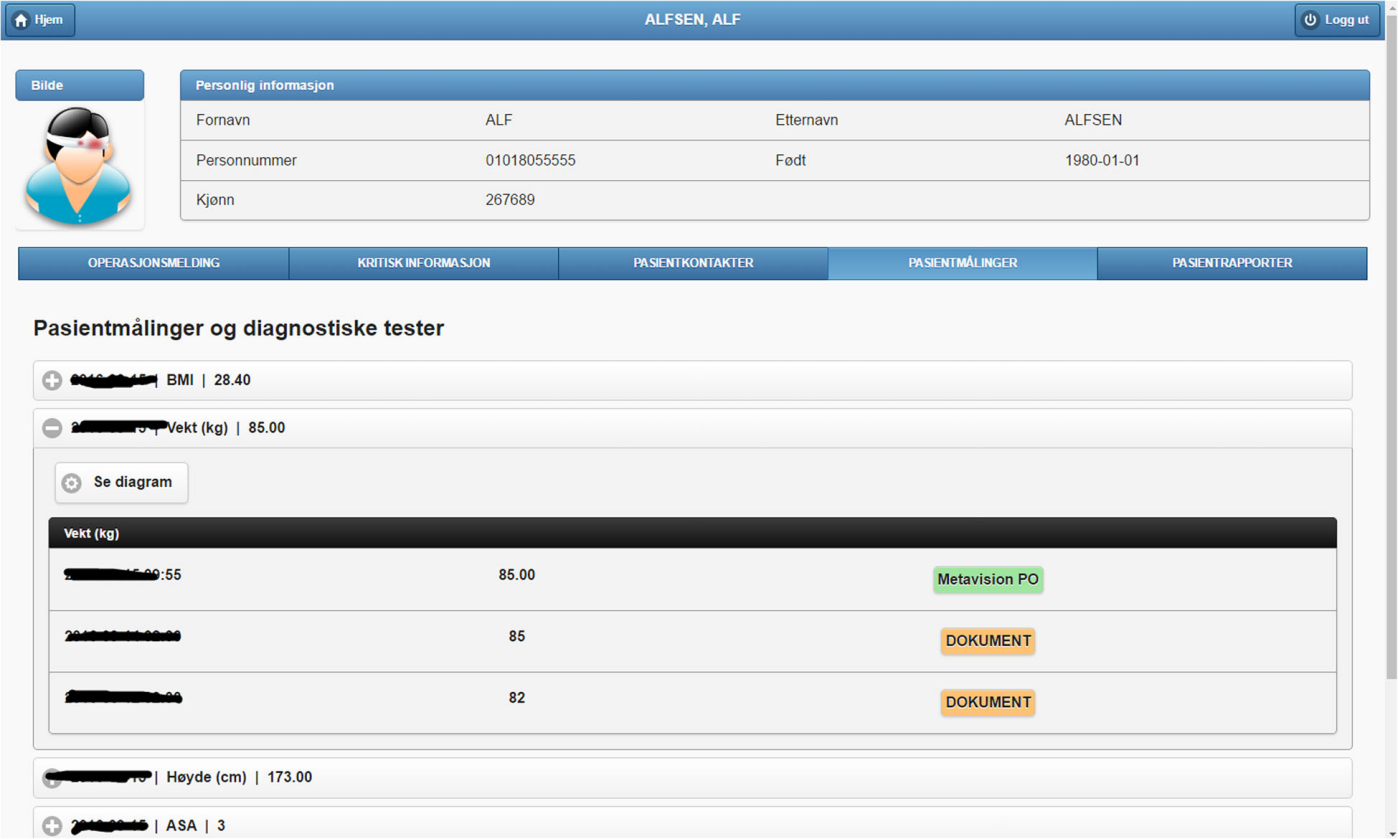
Excerpt of the patient measurements found (tabular mode). The list includes three values of weight measurements obtained from Metavision (1) and documents (2)

**Fig. 5:**
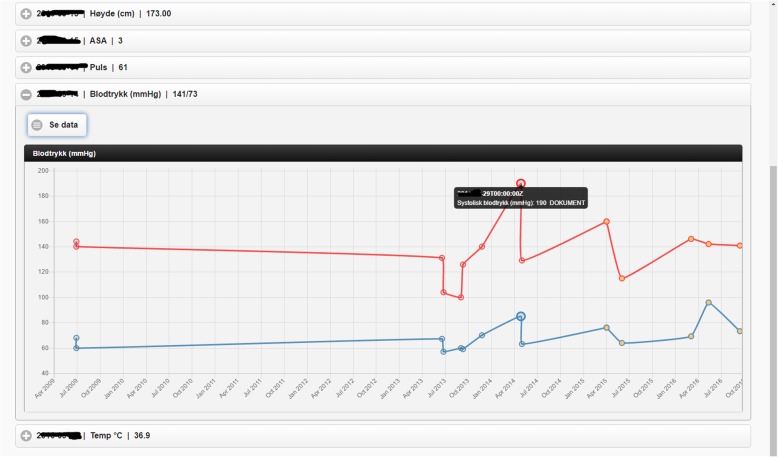
Excerpt of the patient measurements found (graph mode). The graph presents blood pressure data points found in the different sources. The screenshot shows a tooltip of a systolic blood pressure measurement with the timestamp, value and origin

**Fig. 6:**
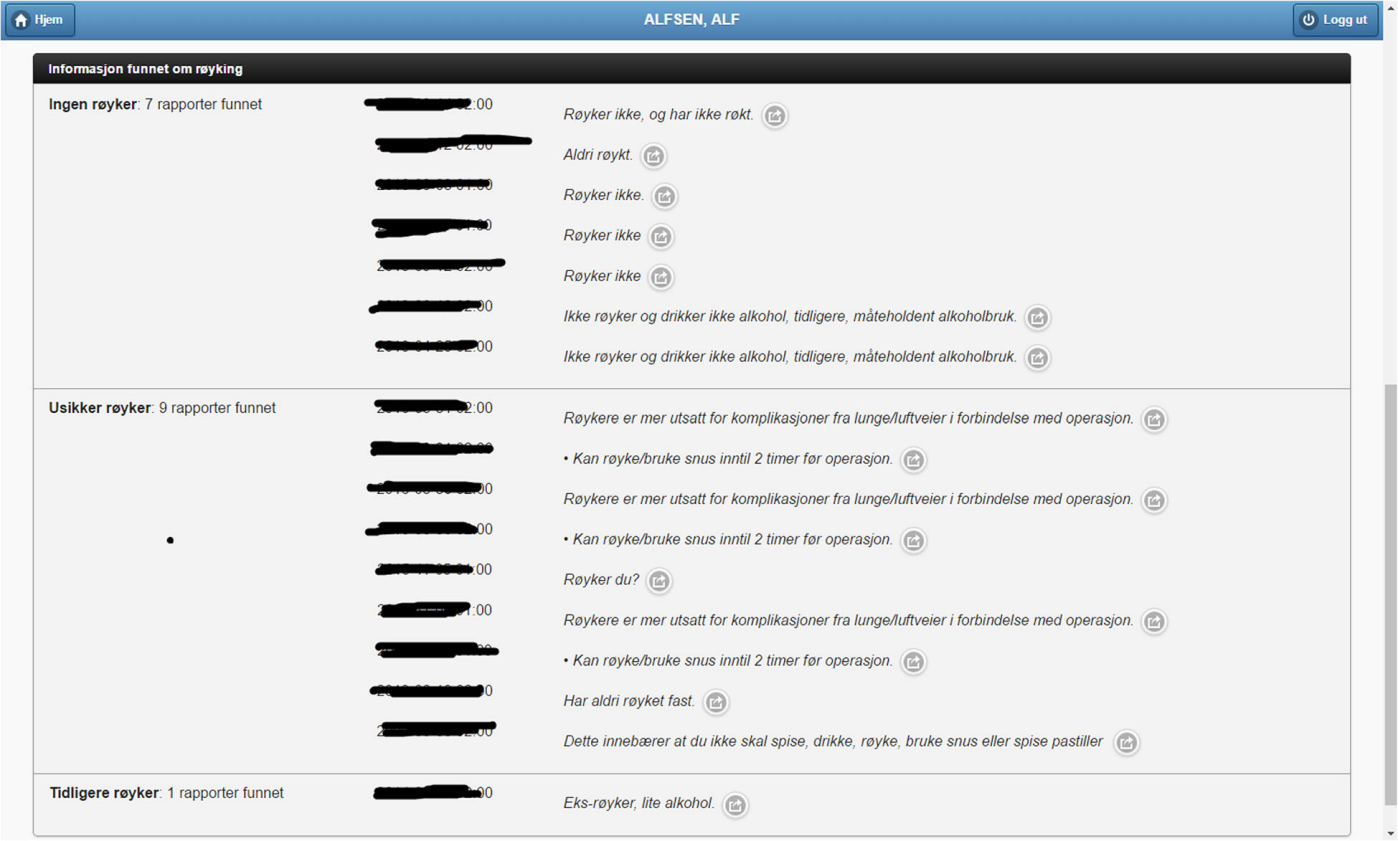
Smoking habits information extracted from the clinical notes of a patient. The list includes 17 occurrences; each one with the predicted smoking status, the document timestamp, the text snippet used for the assessment, and a button to display the whole document

**Fig. 7:**
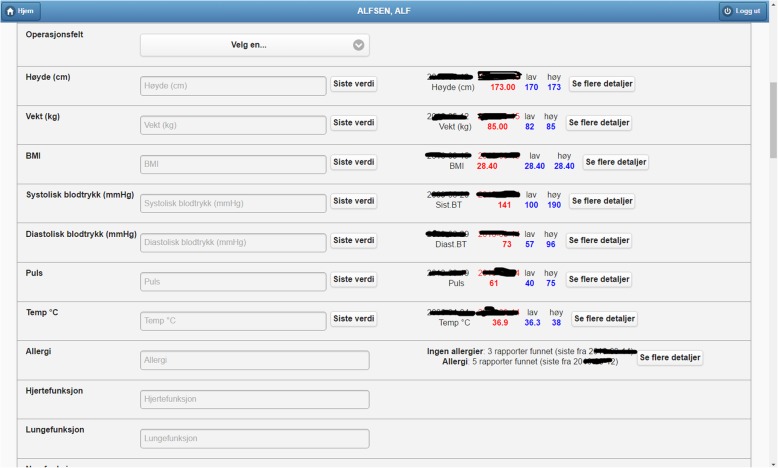
Excerpt of the operation surgery plan form (surgeon section). There are fields for including patient measurements of vital signs, while the right part of the form presents the measures found in the system in a compact way. A summary of patient allergies is also provided

Since the amount of information about a patient can be overwhelming, the GUI is structured in several tabs – see Figs. 2, 3, 4, 5, 6 and 7. Specifically, there is a tab for browsing and querying the clinical notes of a patient; another tab with the patient vital signs; another one with the medical encounters registered in the hospital; another with critical information, e.g. allergies; and a final tab for creating a surgery operation plan. For every data item the GUI provides overviews to limit the amount of information presented, while the user can then zoom in if interested in a particular item.

PreOptique is available with an open source license. The GUI was developed for the Ahus case and can be accessed at [30]. The rest of PreOptique components, i.e. Ontop and Solr, are also available at [31, 32], respectively.

## Results

The ethics committee at Ahus approved this study and 10 patients gave consent to use their health records to demonstrate the system. The IT personnel at Ahus prepared a copy of the production databases using the 10 hospital’s patients and gave us access. We were able to deploy PreOptique and plug in the target data sources, i.e. DIPS, MetaVision and DIPS archive. Specifically, **administrative data** is extracted from DIPS and Metavision; information about **medical encounters** comes from DIPS; and **patient measures** can appear in DIPS and Metavision, but also within clinical documents. As explained in Section 2.4, we exploit text analysis to extract **patient measures** and **information about allergies and smoking habits** from the collection of clinical documents (the latter is not available in any structured dataset at Ahus).

In the remainder of this section we demonstrate the functioning of the system using snapshots taken from this setting. Note that personal patient data has been anonymized and all timestamps removed, and that Ahus approved the use of these snapshots for research purposes.

We organize this demonstrator around the main functionalities of PreOptique, corresponding to the GUI tabs:

1. Medical encounters. The system is able to obtain health patient data coming from DIPS. This is illustrated in Fig. 2, listing the medical encounters of a particular patient in reverse chronological order. The items in yellow correspond to medical processes that are still ongoing. Further details can be obtained by clicking on an item, namely, timestamps, disease label, associated diagnoses and operations.
2. Patient documents. Solr indexes the collection of clinical notes, while the GUI provides controls for browsing and searching the collection – see Fig. 3. Users can type their queries in a text input box. Query expressiveness ranges from simple keyword queries to boolean expressions or fuzzy matches, as supported by Solr. In addition, users can specify date ranges and document types for filtering. As illustrated in Fig. 3, results are displayed in a paginated tabular representation that includes a text snippet and highlights the query terms in yellow. Results are ranked according to query relevance and document freshness. Clicking on the rightmost icon of a table row shows a pop-up window with the whole document.
3. Patient measurements. Measurements of patient vital signs at Ahus are scattered in different sources, including structured databases and annotations in clinical documents. PreOptique is able to retrieve all patient measurements and provide a single and coherent access point, as illustrated in Figs. 4 and 5. In this pilot we extracted the following measurements: BMI, height, weight, illness severity score (ASA), pulse, blood pressure, and temperature. Since there could be many data points for a specific patient, we use collapsibles in the GUI to display the latest value for each measurement type. Expanding a collapsible shows the full list of values in reverse chronological order (see Fig. 4). Users can switch to a graphical representation, as illustrated by Fig. 5. Provenance information is available in the form of buttons (Fig. 4) and tooltips (Fig. 5). Additional details can be obtained by clicking the provenance buttons, e.g. in the case of a document, the document content is shown in a pop-up window with the source text snippet highlighted in yellow.
4. Critical information. This section includes important patient information that should be considered in most medical procedures. In this pilot we only focused on information about allergies and smoking habits, as requested by Ahus. Since this information is missing in the structured databases, clinical notes were the only source employed, applying natural language processing techniques (see section Text Analysis above). Figure 6 shows the information found about the smoking habits of a patient, classified by status. Each section includes a list of the documents found to support the assessment, along with the specific text snippets and timestamps. In this particular example, there are 7 documents classified as “Non smoker”, 9 as “Uncertain smoker”, and 1 as “Ex-smoker”. Interestingly, the snippets of the documents classified as uncertain correspond to smoking questions (do you smoke?), health recommendations about tabaquism, and a protocol for an operation banning smoking. For every document found, the contents can be displayed by clicking the rightmost button.
5. Operation surgery plan. This last tab corresponds to the operation surgery plan that is available as a form structured in three sections – the surgeon completes the first two parts and the anesthesiologist the third one. The first part includes the type of surgery and the resources involved; the second section inquires about the operation details and the patient status from the surgeon’s point of view; and the third part is for the anesthesiologist’s team, requesting information about the patient status, e.g. arrhythmias, asthma, or infarcts. The provided form is identical to the paper-based procedure currently used at Ahus. While this version of PreOptique does not support synchronous editing of the operation surgery plan, the form fields include summaries of existing information found in the system. For example, Fig. 7 shows compact representations of measurements: latest value in red, minimum and highest values, and timestamping information – sparklines are also provided, although Ahus requested to remove them from the snapshots. Users can automatically fill the latest value of a measurement field and they can also browse all the measures using a 1-click button. Similarly, PreOptique presents summaries of the smoking status and allergies of a patient in the corresponding form fields (see the allergy field in Fig. 7). The operation surgery plan is stored in PreOptique and can then be looked up by the medical team (surgeons, anesthetists and nurses) at any time.

Overall, PreOptique seamlessly integrates the data coming from the structured databases and the collection of clinical notes. Patient information is provided in a unified way through a form-based user interface that also supports the preparation of operation surgery plans. As a result, physicians no longer need to access several different systems and manually inspect a collection of documents to check relevant patient information for conducting a particular surgery procedure.

### Preliminary usability study

After the deployment of PreOptique at Ahus, we ran a usability study in order to test the system in the real setting and collect feedback from 5 target users. Their roles were as follows: Senior Medical Advisor, Health Project Manager, Head of Anesthesia, Medical doctor, and Head of Nursing. All of them volunteered and gave their consent to use their study data for research purposes.

Each participant in the study tested the system in a single session lasting 30 to 60 min. No previous training was given, a member of the technical team briefly explained the functionalities to each participant and answered their questions. Participants were requested to complete a questionnaire after testing PreOptique. The employed questionnaire has two sections; the first one corresponds to the System Usability Score (SUS) [33], a popular questionnaire for evaluating the usability of system interfaces. The second part consisted of the following open-ended questions:

- How can this system be employed in your daily practice? [Usage]
- What did you like most about this system? [Like]
- What did you dislike most about this system? [Dislike]
- Other comments and suggestions [Other]

We computed the SUS scores for the participants’ responses, obtaining 86.0 in average with a standard deviation of 10.7.^1^ Since we were not allowed to use test versions of DIPS and Metavision in this usability study, we employ historical SUS scores from other studies for comparison purposes ([34], ch. 8). Reports SUS statistics based on data from 446 studies and over 5000 individual SUS responses, finding that the mean SUS score is 68 and the 97% percentile rank is 85. Therefore, the obtained SUS score of 86.0 can be considered high.

Beyond the SUS results, we analyzed the responses to the second part of the questionnaire, extracted the topics, and compiled the results displayed in Table 2. Participants in the study stressed the integration of patient data as the principal advantage of PreOptique. They also considered the system to be easy to use and fast. About the limitations, one participant indicated that the security functionality is not completely in place, and there were several comments stressing the need to move to the production stage.

**Table 2:** Main findings obtained from the usability questionnaire along with supporting statements from participants’ responses and source (question type and participant code)

Finding	Sample comments	Source
Enables patient data integration	*Perfect to find information that now is “hidden” because you don't have time to seek*	[Usage - P1]
	*The fast integration of patient information/data from different systems, incl. text mining*	[Like - P3]
	*Gather info from different databases*	[Usage - P5]
Fast	*Fast to find information*	[Like - P2]
Easy to use	*It gives information that I need very easy*	[Like - P1]
	*Easy to find patient stats*	[Like - P4]
	*Easy to understand*	[Like - P5]
Lacks some security features	*Security functionality not completely in place (work in progress)*	[Dislike - P2]
Transition to production deployment	*Full roll-out as soon as possible*	[Other - P2]
	*It is not yet ready to use*	[Dislike - P1]
	*Only a small demo*	[Dislike - P5]
	*Looking forward to next step*	[Other - P5]

## Discussion

### Principal results

Data integration is a hard problem that involves a number of challenges that can be grouped into system-based, logical, and, social categories ([35], ch. 1). System-based challenges must address methods for enabling different systems to communicate seamlessly to one another. Logical challenges involve correcting for differences in the structure of the data sources, e.g. different schemata. Social challenges cover a number of non-technical problems such as data owners not wanting to cooperate. The PreOptique system exemplifies how OBDA technology can provide integrated data access to disparate structured sources in healthcare. Patient data integration is achieved through the specification of an ontology and mappings to the source databases. Furthermore, this solution relies on open standards and does not require the replacement of existing databases, thus dramatically reducing the installation overhead.

While it is possible to design an SQL-based solution to integrate DIPS and Metavision, an ontology is a sensible conceptual middle layer between differing data models and the non-technical end users. In describing the domain, instead of the data, an ontology provides a common layer over differently modelled databases. As a matter of fact, the OBDA approach provides several advantages over a pure SQL-based solution [3]: (1) data integration is performed declaratively through a system-independent specification of the domain, i.e. the ontology; (2) physical/logical independence of the information system is further enforced, thus improving data access by non-experts; (3) data integration can be carried out in an incremental way; and (4) the ontology provides a common ground for the documentation of the data sources. The proposed ontology not only can be applied to the Ahus case, but to other surgical units in Norway since DIPS and Metavision are deployed in every hospital in this country.

PreOptique also exploits unstructured clinical text by using a search engine and natural language processing for extracting patient findings. The GUI provides a single access point and hides the underlying complexity of the system. A form-based interface is employed to present patient data, while user actions are translated into SPARQL and Solr queries behind the scenes. The ontology enforces the coherence of patient data, using terms from the medical domain. Participants in the usability study stressed the simplicity of the GUI and the integration of data sources that the system enables, while the obtained SUS score was quite high.

Importantly, PreOptique is designed to improve access to patient data, but not to make medical decisions. As a result, the main focus is to pull data from different sources, to present the integrated data points in a consistent way, and to give access to the source data (including provenance information). Participants’ comments in the usability study suggest that the system can be effective for finding relevant patient information in a timely way – see Table 2.

### Limitations

We were only allowed to test the system with a copy of the production databases for 10 patients at Ahus. Nevertheless, this should not entail scalability issues, since query complexity does not change with the number of patients. Some of the patients have hundreds of clinical documents associated with numerous medical encounters. Given that PreOptique queries patient data on demand and caches responses, perceived performance was good, as pointed out by participants in the usability study. Furthermore, Optique has been successfully tested in the data-intensive petroleum company Statoil [11]. This use case is far more complex than the one considered here, since the EPDS dataset alone (Statoil’s central repository of exploration and production data) has about 3 K tables, ~ 37 K columns all together, and a size of ~ 700 GB. Experience from Statoil showed that the time required to generate SQL queries through ontology mappings was negligible compared to query execution time. About the efficiency of the generated SQL queries, running times were no worse than what a database expert could have achieved manually [11]. Nonetheless, we plan to carry out a performance study with a larger patient base in order to assess whether PreOptique can be used to examine patient data without performance issues, as well as to respond to ad hoc queries such as the one listed in Section 2.4.

The scope of this pilot was restricted to the surgery operation planning, and some data sources such as laboratory tests or patient medications were explicitly excluded by the project management for this pilot. Thus, a natural extension point is the integration of the aforementioned databases. In this way, PreOptique can better support surgery operation planning and can be easily extended to support other hospital processes such as medication orders.

While the number of participants in the usability study is relatively low, experts in the field report that only five participants are needed on average to find 85% of usability problems in a design [36]. Further, [37] found no correlation between the number of usability problems detected and the number of participants (ranging from 5 to 15) when evaluating the same interface design. In any case, we do not make claims about the statistical significance of the results, since our analysis is rooted in the qualitative research tradition [38].

### Comparison with prior work

Integration of patient data is quite challenging due to the range of different data types [15] and the diversity of medical information systems involved [39, 40]. As a result, there is a plethora of standards, terminologies and initiatives for interoperability in healthcare [15]. For example, OpenEHR [41] is an open architecture designed to support the development of distributed patient health records. One of the main problems for the adoption of interoperability standards is handling legacy systems and data [42]. In this regard, our solution (and OBDA in general) does not assume any particular standard or data format; the mappings are designed to address the mismatch between the ontology and the underlying data sources.

There have been several initiatives trying to use ontologies to solve interoperability problems in healthcare, but only a few propose a system implementation, i.e. an OBDA system. For example, [43] uses ontologies to transfer medical records from structured databases to an RDF triple store. This solution adopts the materialization approach, typical of data warehouses, that has several drawbacks, namely, data duplication, need for synchronization, and potentially slow query execution, although materialization allows quite flexible transformations of data. In contrast, PreOptique uses query rewriting that does not require data duplication and synchronization, despite the fact that transformations are restricted to the query language supported by the sources and that transformations are performed at query time.

Existing OBDA systems in the medical domain assume that end users will employ a formal query language like SPARQL – this is the case of [43, 44]. However, this is a very stringent constraint that cannot be met in many settings, as in the Ahus case. For this reason, we developed a form-based GUI to easily interact with the system instead of providing users with a query editor. While this GUI was purposely designed to support the functionalities of the Ahus case, a generic visual query editor like OptiqueVQS [6] or PepeSearch [7] can also be employed to formulate ad hoc queries by the medical staff, e.g. to find the set of patients with a particular disease.

Extracting information from clinical text is a difficult problem, and there is a whole body of research in this field – see for instance [45]. Here, our focus was not the proposal of novel natural language processing techniques, but the integration of clinical text as another data source. This was addressed through the GUI component, since the Optique platform (and OBDA in general) is purposed for structured databases.

## Conclusions

Providing medical staff with flexible access to patient data is a major bottleneck in the healthcare domain. In this pilot study with Ahus, we showcased a hybrid semantic and text-based system, PreOptique, enabling the integration of disparate data sources. We developed an ontology for capturing nurses’ and doctors’ vocabulary and prepared a set of mappings from the ontology to the structured databases. Clinical notes were also indexed and analyzed to extract patient information. We designed a form-based GUI to access patient data and to support surgery operation plans, as requested by Ahus. PreOptique was successfully deployed at the hospital, enabling the seamless integration of patient data. Results from a preliminary usability study suggest that medical personnel can easily find patient information in a timely way. Our future work includes the integration of new databases such as patient medications and laboratory tests; further user studies, including a formal experiment for gathering the critical information for surgery procedures; a performance experiment to test PreOptique with a larger patient base; and the deployment of PreOptique in the production IT environment at Ahus.

## Availability and requirements

Project name: PreOptique

Project home page: https://github.com/guiveg/preoptique

Operating system: Platform independent

Programming language: JavaScript

Other requirements: Ontop, Solr 6

License: Apache 2.0

## Abbreviations

Ahus: Akershus University Hospital
EHR: Electronic Health Record
GUI: Graphical User Interface
IT: Information Technology
NLP: Natural Language Processing
OBDA: Ontology-Based Data Access
SQL: Structured Query Language
SUS: System Usability Score

## Footnotes

1 SUS scores range from 0 to 100.
